# Identification and characterization of Iporin as a novel interaction partner for rab1

**DOI:** 10.1186/1471-2121-6-15

**Published:** 2005-03-29

**Authors:** Michael Bayer, Julia Fischer, Joachim Kremerskothen, Edith Ossendorf, Theodoros Matanis, Magdalena Konczal, Thomas Weide, Angelika Barnekow

**Affiliations:** 1 UKM Muenster, Albert-Schweitzer-Str. 33, D-48149 Muenster, Germany; 2 Department of Experimental Tumorbiology, University of Muenster, Badestr. 9, D-48149 Muenster, Germany; 3 Cilian AG, Johann-Krane-Weg 42, D-48149 Muenster, Germany

## Abstract

**Background:**

The small GTPase rab1a and its isoform rab1b are essential regulating components in the vesicle transport between the ER and the Golgi apparatus. Rab1 is thought to act as a molecular switch and can change between an active GTP-bound and an inactive GDP-bound conformation. To elucidate the function of rab1, several approaches have been established to isolate effector proteins, which interact with the activated conformation of rab1. To date p115, GM130, golgin-84 and MICAL have been identified as direct interacting partners. Together with rab1, these molecules are components of a protein complex, which mediates and regulates intracellular vesicle transport.

**Results:**

Here, we report the characterization of Iporin, which is similar to KIAA0375 as a novel rab1-interacting protein. It was initially identified by yeast two-hybrid screening experiments with the active mutant of rab1b (rab1b Q67R) as bait. Iporin contains a SH3 domain and two polyproline stretches, which are known to play a role in protein/protein interactions. In addition, Iporin encloses a RUN domain, which seems to be a major part of the rab1binding domain (R1BD). Iporin is ubiquitously expressed and immunofluorescence staining displays a cytosolic punctual distribution. Interestingly, we also show that Iporin interacts with another rab1 interacting partner, the GM130 protein.

**Conclusion:**

Our results demonstrate that Iporin is a potential new interacting partner of rab1. Iporin is different from already identified rab1 interacting proteins concerning protein structure and cellular localization. We conclude that Iporin might function as a link between the targeting of ER derived vesicles, triggered by the rab1 GTPase and a signaling pathway regulated by molecules containing SH3 and/or poly-proline regions. The characterization of this novel intermolecular relation could help to elucidate how vesicles find their way from ER to the Golgi apparatus.

## Background

The rab/Ypt proteins represent a large family of small GTPases involved in several transport steps of the cellular trafficking pathway. At present 11 members have been identified in yeast and more than 60 human rab proteins are supposed [1,2]. Small GTPases are known to function as molecular switches and can cycle between an active GTP-bound and an inactive GDP-bound conformation [3,4]. In the active conformation, the rab GTPases are localized on membranes, where they recruit specific effector molecules to mediate vesicle attachment [5]. The subsequent fusion step is initiated, when the membranes are forced into close proximity by the interaction of SNARES (soluble N-ethylmaleimide-sensitive factor attachment protein receptor) presented on both membranes in concert with SM proteins (Sec1/Munc18-like proteins), thought to act as controlling elements in SNARE interaction [6].

The two isoforms of the small GTPase rab1 (rab1a/b) are localized on ER-Golgi membranes and regulate the exocytotic transport from the ER to the Golgi apparatus [7-9]. Although the function of rab1 is still not fully understood, several lines of evidence describe rab1 and its yeast homolog Ypt1p GTPase as a major component in a protein complex responsible for directing vesicular traffic between the ER and the Golgi apparatus [7-13]. In the past, several approaches have been established to identify effector molecules that bind specifically to the active GTP-bound form of rab1 [14-17]. At present, four specific effector proteins and several additional putative effector molecules are known [14]. The first characterized rab1 interacting protein was p115, which also binds to the Golgi-associated proteins GM130 and Giantin [14,18,19]. p115 is the mammalian homologue of the yeast protein Uso1p, which was found to be an essential component necessary for the tethering of ER-derived COP II vesicles to the Golgi compartment, a process that depends on Ypt1p, but is independent of SNARE proteins [20]. In addition, p115 plays a key role in coordinating sequential tethering and docking of COPI vesicles to Golgi membranes. Thus, it is thought to function as a linker protein between Giantin and GM130 [19,21,22]. Second, the cis-Golgi matrix protein GM130 was identified as a direct rab1 effector, which also binds to a complex containing GRASP65, a lipid-anchored GM130-binding protein, that regulates Golgi stack reformation following mitosis, as well as so far uncharacterized proteins [15,17,23,24]. A third interacting molecule of activated rab1, golgin-84, is supposed to act as a novel mitotic target [16]. Overexpression or depletion of golgin-84 results in fragmentation of the Golgi ribbon and the protein is thought to be required for the incorporation of membranes into the Golgi apparatus [16,25]. Golgin-84 is a type II C-terminally anchored cis-Golgi protein with an extensive cytoplasmic coiled-coil domain, which shows structural and sequence similarity to Giantin [26]. However, golgin-84 does not appear to physically interact with other cis-Golgi matrix proteins such as GM130, p115 or GRASP65 and therefore it is likely, that additional potential golgin84-binding molecules exist [16]. Recently, we identified MICAL as a fourth rab1 interacting partner and suggested a link between the Golgi apparatus associated rab1 and the intermediate filament cytoskeleton [27].

Here, we describe Iporin as a novel interacting molecule for the active GTP-bound conformation of rab1. Iporin (Interacting protein of rab1) was identified in a yeast two-hybrid screen with rab1b Q67R as bait and sequence analyses revealed, that the encoded protein is similar to the C-terminal half of the KIAA0375 polypeptide. The rab1b-Iporin interaction is nucleotide-dependent and rab1-specific. Interestingly, the rab1 binding motif of Iporin was mapped to a region containing a RUN domain, a protein motif, which is known to be involved in the function of small GTPases. Furthermore we examined the intracellular localization and tissue-specific expression of Iporin and give insight into its domain structure. Interestingly our results also showed an interaction with the rab1 effector GM130 via its coiled-coil region 6 [15].

## Results

### Identification of a novel rab1 interacting protein

With the rab1b Q67R mutant as bait and a human placenta cDNA library as prey we performed yeast two-hybrid screening assays to identify novel interacting partners for the active GTP-bound conformation of the small GTPase rab1b. One of the isolated clones could be identified as the human Golgi matrix protein GM130 and a second clone encoded for a fragment of MICAL1b [15,27]. Another interesting clone was K17, which displayed a strong and specific interaction with rab1b Q67R in the yeast two-hybrid system (Table 1). Nucleotide sequence analysis revealed that K17 represents the cDNA sequence encoding the C-terminus of KIAA0375 (GenBank Accession number: AB002373.2). We named the identified protein Iporin (for Interacting protein of rab1) and the deleted K17 clone Iporin ΔN847. Since the rab1 effector GM130 has been shown to interact with various rab proteins (rab1, rab2, rab33b), we wanted to investigate the rab1 specificity of the Iporin interaction [16,23,28]. Thus, we used an assortment of rab cDNA molecules as bait constructs in yeast co-transformation assays (Table 1). Iporin ΔN847 (K17) induced strong growth and β-galactosidase activity only, when co-transformed with the permanent active mutants of rab1b (rab1bQ67R/L) and rab1a (rab1a Q70L).

**Table 1:** Interaction of Iporin ΔN847 with different rab proteins/isoforms

	**prey Iporin ΔN847**		
**bait**	control	his3	lacZ
pAS2-1	+++	-	-
		
rab1a wt	+++	+	+/-
rab1a S25N	+++	-	-
rab1a Q70L	+++	+++	+++
		
rab1b wt	+++	+/-	-
rab1b S22N	+++	-	-
rab1b Q67R	+++	++	+++
rab1b Q67L	+++	++	+++
		
rab2 wt	+++	-	-
rab2 S20N	+++	-	-
rab2 Q65L	+++	-	-
		
rab6 wt	+++	-	-
rab6 T27N	+++	-	-
rab6 Q72R	+++	-	-
		
Ypt1 wt	+++	++	++
Ypt1 S22N	+++	-	-
Ypt1 Q67L	+++	+++	+++

Results obtained from co-transformation assays in yeast. Clones were cultivated on selection plates lacking tryptophan and leucine (control) or lacking additionally histidine and were supplemented with 50 mM 3-aminotriazol (his3). The β-galactosidase activity was determined using X-gal as substrate on filter lift assays (lacZ). - = no growth on selection plates or no β-galactosidase activity; +/- = background growth or β-galactosidase activity appears overnight; + = slow growth; ++ = strong growth or β-galactosidase activity appears after a few hours; +++ = very fast growth or high β-galactosidase activity.

Interestingly, an interaction between Iporin ΔN847 and the wild type as well as the permanently active mutant of the yeast counterpart of mammalian rab1, Ypt1p, was observed pointing to a conserved interaction of these two molecules [5]. The empty pAS2-1 vector, the wildtypes of the rab1 isoforms (rab1a/b), permanently inactive mutants of the rab1a/b (rab1a S25N, rab1b S22N) and Ypt1p (Ypt1 S22N) proteins were not able to significantly activate reporter gene expression (Table 1). There were also no interactions of IporinΔN847 with the small GTPases rab6 and rab2 (Table 1). These results indicate, that the interaction with Iporin ΔN847 is rab1-specific and requires the GTP-bound, active conformation of rab1.

### Mapping the Iporin binding site of rab1b

**Figure 1:**
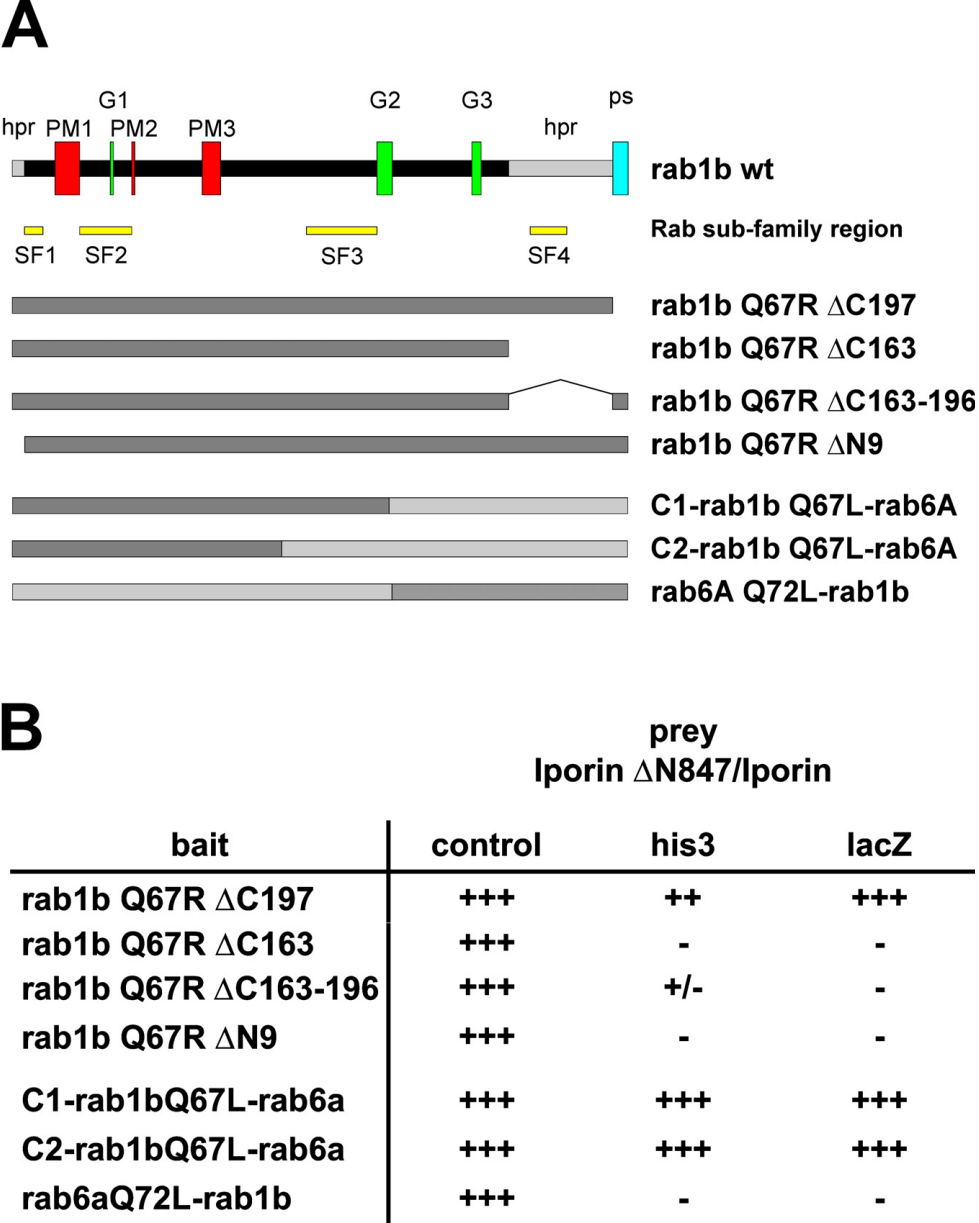
**Determination of the Iporin interacting domain. (A) **Schematic overview of the domain structure of rab1b with the conserved G1-G3 boxes, PM1-3 motifs, N-terminal and C-terminal hypervariable regions (hpr) and the C-terminal prenylation site (ps). SF1-4 indicate the rab subfamily regions. The rab1b regions of the chimeras are shaded dark and the rab6 regions are light-shaded. **(B) **All constructs were co-transformed in yeast and cells were cultivated on selection plates as described above (Table 1). Rab1b deletion mutants were co-transformed with Iporin ΔN847, whereas chimeras were co-transformed with full-length Iporin. - = no growth on selection plates or no β-galactosidase activity; +/- = background growth or β-galactosidase activity appears overnight; ++ = strong growth; +++ = very fast growth or high β-galactosidase activity

Next, we wanted to analyze, which structural elements of rab1b are necessary or sufficient for the interaction with Iporin. Since we already demonstrated that the hypervariable regions of rab1 are necessary for an interaction with GM130, we created rab1b mutants as well as rab1/rab6 chimeras (Figure 1A) [15,29]. The mutants and the chimeras enabled us to restrict specific regions for the interaction to the C- or N-terminal half of rab1. Co-transformation experiments in yeast revealed that only the rab1b deletion mutant rab1b Q67R ΔC197 lacking the prenylation site of rab1b showed a strong interaction with Iporin ΔN847, suggesting that the prenylation site is not necessary for the interaction with Iporin ΔN847 (Figure 1B). The deletion mutants rab1b Q67R ΔN9, rab1b Q67R ΔC163 and rab1b Q67R ΔC163-196 failed to interact with Iporin ΔN847 (Figure 1B). These mutants were constructed based on the model previously proposed by Bourne *et al*. and Valencia *et al*.. G1-G3 and PM1-PM3 are conserved regions in all members of the ras superfamily of GTPases, that are involved in guanine (G) and phosphate/Mg^2+ ^(PM) binding, respectively [3,4,30]. According to the proposed model suggested by Seabra and coworkers, which defined rab conserved sequences as rab family motifs (RabF1-5) and other regions as subfamily motifs (RabSF1-4), the generated mutants lack the RabSF1 or RabSF4 region (Figure 1A) [31]. Co-transformation with the two rab1/rab6 chimeras displayed an interaction between the C1- and C2-rab1b Q67R-rab6a chimera and Iporin/Iporin ΔN847, but not between the rab6a Q72L-rab1b chimera and Iporin ΔN847 (Figure 1B).

### Full-length cloning and characterization of Iporin

Sequence analysis of the clone K17 cDNA revealed an open reading frame of 669 amino acids, which was identical to the C-terminal half of the protein encoded by the KIAA0375 cDNA (aa 848–1516). To obtain the full-length Iporin sequence, we used the KIAA0375 cDNA for the amplification of the sequence encoding the missing N-terminal fragment by PCR.

**Figure 2:**
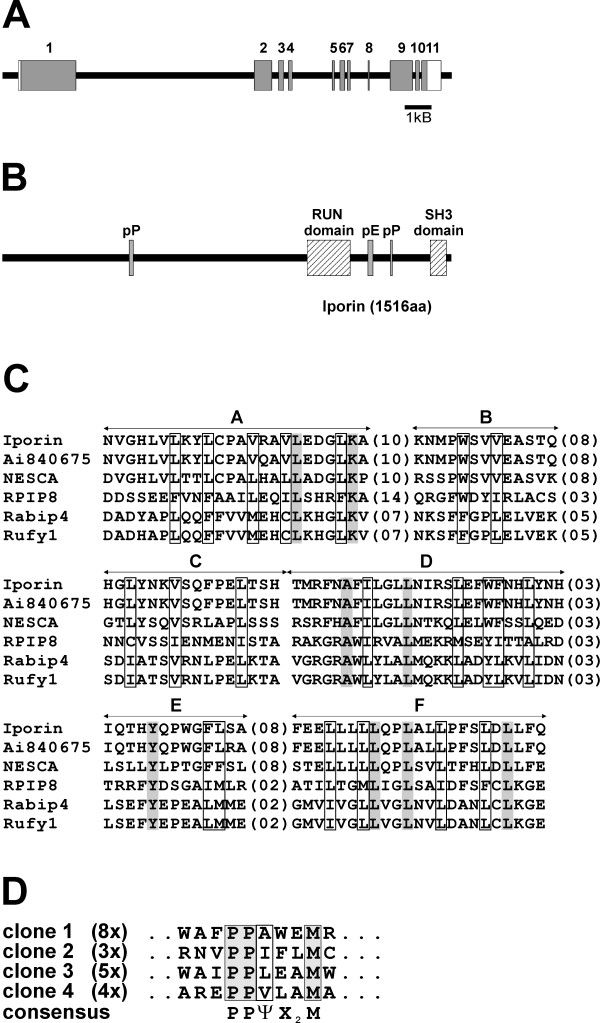
**2 Genomic structure and functional elements of Iporin. (A) **Schematic representation of the human Iporin gene located on chromosome 9. The protein coding exons of Iporin are shown as shaded boxes numbered one to eleven, non-coding sequences are white. The gene (~14 kb) encodes a protein of 1516 amino acids. **(B) **Domains and structural motifs of Iporin. The most interesting domains within Iporin are the SH3 (aa 1447–1500) and the RUN domain (aa 1031–1175). The proline-rich motifs (pP, aa 429–442; aa 1311–1319) are possible interaction motifs for SH3 or WW domain containing proteins. The glutamic acid-rich region (pE, aa 1236–1252) is negatively charged with possibly stabilizing function. **(C) **Alignment of RUN domains from related proteins. The sequence corresponding to the RUN domain of Iporin (aa 1031–1175, human) was aligned to the RUN domains present in AI840675 (aa 309–453, mouse), NESCA (aa 53–197, human), RPIP8 (aa 52–189, human), Rabip4 (aa 31–163, mouse) and Rufy1 (aa 31–163, human) (GenBank accession numbers, XP_131380.2, BAA77507.2, NP_006686.1, CAC17732.1 and AAK50771.1, respectively). RUN domains were detected using the Motif Scan software . Hydrophobic residues (V, L, I, F, Y, W, M, C) are show as boxes, conserved positions are shaded. A-F are the six conserved blocks, and distances between these blocks are indicated by the numbers of residues in parentheses. **(D) **Alignment of sequenced clones identified by phage display using the Iporin SH3 domain. Four different clones were isolated, whereby the number of independent clones, which contained the same sequence, are in brackets. The conserved consensus motif is shaded. Ψ = aliphatic amino acid; X_2 _= two individual amino acids.

Information about the genomic structure of Iporin protein was obtained from the human genome database. The Iporin gene consists of ~14 kb of genomic sequence and contains eleven exons (Figure 2A). The gene is located on the short arm of chromosome 9 (9p13.1) and comprises 4548 bp of coding DNA. Deduced from the cDNA sequence, Iporin comprises 1516 amino acids with a calculated molecular mass of 161.195 kDa and an acidic pI of 6.17. Further structure analysis revealed functional domains and motifs in the Iporin sequence (Figure 2B). Iporin contains some low complexity regions, which include polyproline (pP) and polyglutamic acid (pE) stretches. The first polyproline stretch is localized between aa 429 and aa 442 with the sequence PPPGPGPDPGPSQP and a second region between aa 1311 and aa 1319 with the sequence PPQAPPP. Proline-rich stretches are known to interact with SH3 or WW domains and could mediate further protein/protein interactions [32]. The polyglutamic acid stretch, spanning aa 1236 – 1252 with the sequence EGGEEEEEEEETEEVAE is flanked by the two proline-rich regions. This motif is highly negatively charged and may play a role in stabilizing the protein or function as further interaction domain.

Using the Motif Scan Software  we also detected a RUN (RPIP8, UNC-14 and NESCA) domain in the Iporin sequence (aa 1031–1175; Figure 2B). This domain was shown to be involved in the function of small GTPases and can also be found in proteins which play a role in proliferation, differentiation and motility [33,34]. The RUN domain is organized in six conserved blocks (Figure 2C; A-F), which are predicted to constitute the "core" of a globular structure. Figure 2C shows an alignment of different RUN domains containing proteins related to small GTPases like RPIP8, Rabip4, Rufy1, NESCA or Iporin [34-36]. RUN domain containing proteins enclose hydrophobic amino acids in conserved positions as shown by shaded or boxed regions (Figure 2C). The secondary structure of the RUN domain core was predicted to consist of predominantly α-helices and the conserved three dimensional structure is probably of importance. However, the exact function of this motif is still unclear [33].

Interestingly, NESCA (new molecule containing SH3 at the carboxy-terminus) and Iporin share 42% of sequence homology and contain both a SH3 domain at the C-terminus. The function of NESCA is not known, but it is thought to be involved in signal transduction pathways and was recently described as a signaling adaptor which shuttles from the cytosol to the nuclear envelope [37,38]. We used NESCA as a prey for yeast two-hybrid assays to investigate, whether NESCA might be a further interacting partner of the small GTPase rab1. However, we were not able to show an interaction between NESCA and rab1 (data not shown).

BLAST searches revealed a mouse homolog of Iporin, EST clone AI840675, which shows 84% similarity to the human protein. This clone encodes 736 amino acids, which are homologous to Iporin aa 723 to 1455. The truncated mouse Iporin protein contains only a RUN domain and we suppose that it might function in a different, but related manner, to human Iporin. The C-terminus of Iporin contains a SH3 domain (aa 1447–1500), which is found in a variety of proteins (Figure 2B) [39]. This domain is known to interact with polyproline domains in target molecules and is involved in transmembrane signaling and cytoskeletal rearrangements [40]. To identify the interacting motif of the SH3 domain of Iporin we performed a phage displayed analysis. For this purpose we screened a fUSE5/15-mer M13 phage displayed random peptide library using purified GST-Iporin SH3 (Iporin ΔN1430) as a target. Sequencing of the cDNA from phages with a high and specific affinity towards GST-Iporin SH3 identified a new consensus motif (Figure 2D). This motif contains two prolines followed by an aliphatic amino acid (A, I, L, V), two further amino acids and a conserved methionine (PPΨX_2_M).

### Mapping the rab1 binding domain of Iporin

**Figure 3:**
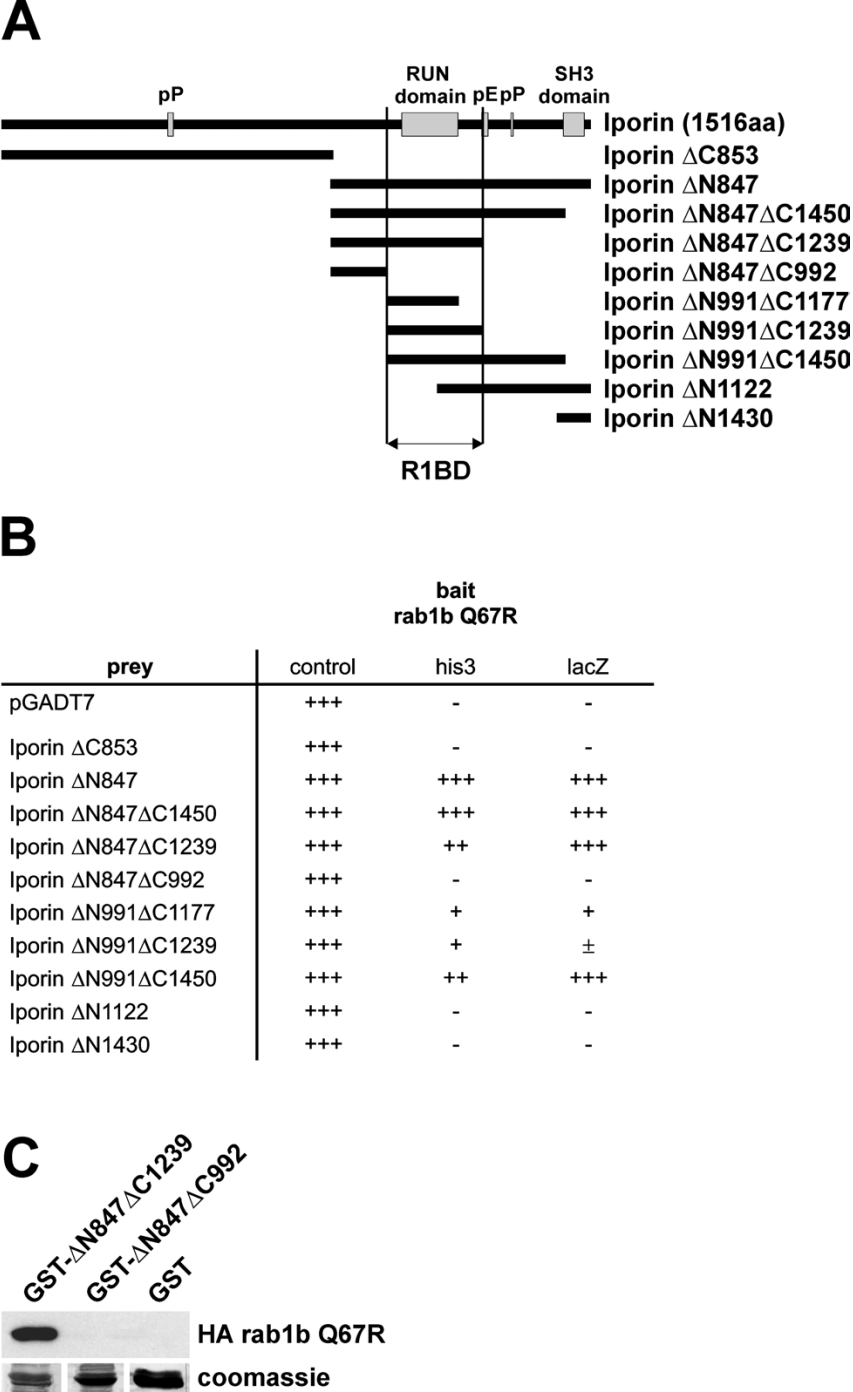
**Mapping the rab1 binding domain (R1BD) of Iporin. (A) **Overview of deletion mutants used for yeast co-transformation assays. The deletion mutants ΔNx or ΔCx do not contain the N-terminus or the C-terminus, respectively. We were able to map the rab1 binding domain (R1BD) to a RUN domain containing region. **(B) **Results obtained from yeast co-transformations. The bait construct rab1b Q67R was cloned into the pAS2-1 vector and the prey constructs into pGADT7. Clones were cultivated on selection plates as described previously (see Table 1). **(C) **GST-pulldown with coupled GST-Iporin ΔN847ΔC1239 indicated an interaction with Iporin, whereas GST-Iporin ΔN847ΔC992 and GST were negative. The lower panel shows the Coomassie blue stained blot membrane with equal amounts of GST fusion protein. - = no growth on selection plates or no β-galactosidase activity; +/- = β-galactosidase activity appears overnight; + = slow growth or β-galactosidase activity appears after several hours; ++ = strong growth; +++ = very fast growth or high β-galactosidase activity; pP = polyproline; pE = polyglutamic acid.

To investigate, which part of the Iporin sequence acts as a rab1 binding domain (R1BD), we generated several deletion mutants of Iporin (Figure 3A) and tested them in yeast two-hybrid assays (Figure 3B). As expected, the N-terminus of the protein (Iporin ΔC853), which is not present in the original prey clone as well as the SH3 domain (Iporin ΔN1430) are not necessary for the interaction with rab1b and failed to activate the reporter genes (Figure 3B). Only the RUN domain-containing mutants (Iporin ΔN847, ΔN847ΔC1450, ΔN847ΔC1239, ΔN991ΔC1177, ΔN991ΔC1239 and ΔN991ΔC1450) showed growth and β-galactosidase activity on selection plates (Figure 3B). Interestingly, the interaction of rab1b with the shortest RUN domain containing mutants (ΔN991ΔC1177, ΔN991ΔC1239, respectively) was weaker compared to fragments containing longer flanking sequences, which could result from protein misfolding.

To confirm the yeast two-hybrid mapping results, we performed GST-pulldown assays. Bacterially expressed GST-Iporin ΔN847ΔC1239, GST-Iporin ΔN847ΔC992 and GST were coupled to glutathione-Sepharose and incubated with extract from BHK cells overexpressing HA-tagged rab1b Q67R (Figure 3C). In this assay, the GST-Iporin ΔN847ΔC1239 was able to bind to the active form of rab1b, whereas GST and GST-Iporin ΔN847ΔC992 failed to interact (Figure 3C). These findings confirmed the yeast data (Figure 3B) and indicate that the rab1 binding domain of Iporin needs the whole RUN motif.

### Tissue-specific expression and cellular localization of Iporin

**Figure 4:**
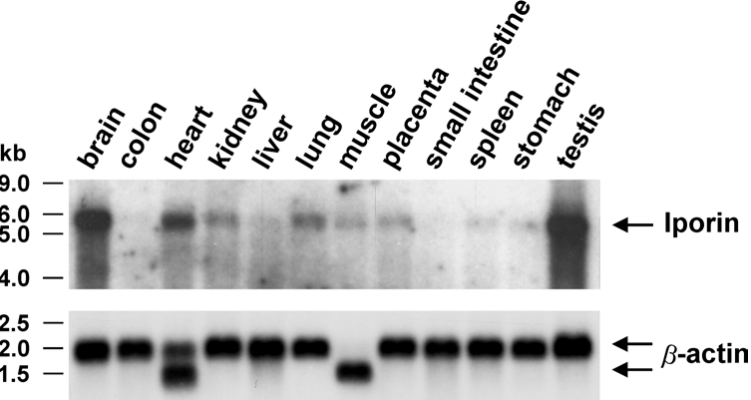
**Tissue-specific expression of Iporin mRNA. **A 12-lane multiple-tissue Northern blot was hybridized with a specific ^32^P-labeled cDNA probe for Iporin. Each lane contains approximately 2 μg of poly A^+ ^from various tissues. The blot was exposed to an X-ray film for 17 h at -80°C with an amplifying screen. To confirm equal loading, the membrane was re-hybridized with a radiolabeled probe specific for β-actin mRNA.

Northern blot analysis of various human tissues indicated, that Iporin is ubiquitously expressed. The Iporin mRNA was identified as a prominent band of approximately 5.5 kb. Highest amounts of Iporin transcripts were observed in brain and testis (Figure 4). These findings are consistent with the RT-PCR results, published by the Kazusa institute, Japan.

The cellular distribution of Iporin was examined by cytosol/membrane fractionation and immunofluorescence.

**Figure 5:**
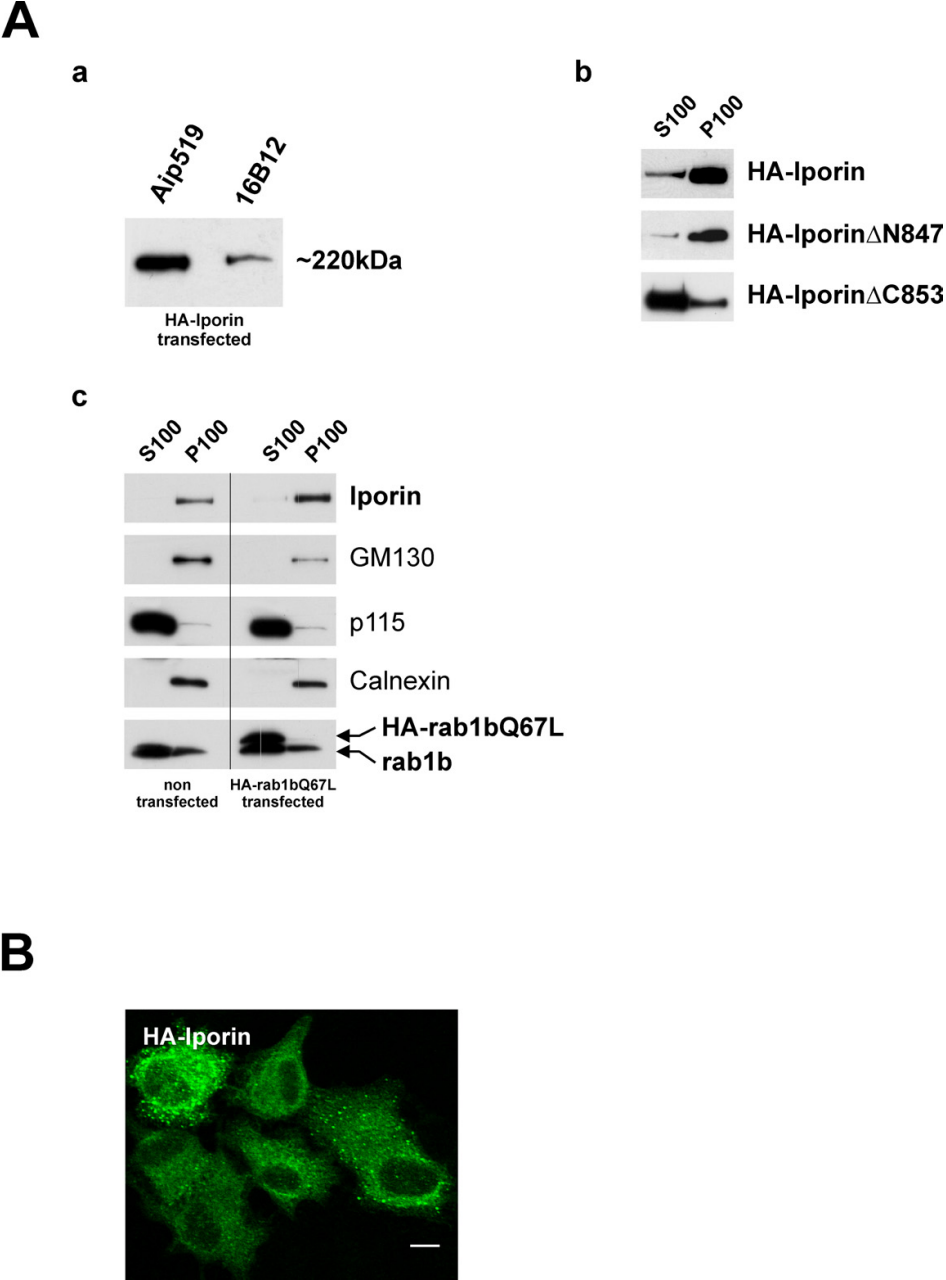
**Cellular distribution of Iporin. (Aa) **To test specificity of the polyclonal anti Iporin antibody Aip519, HeLa cells were transiently transfected with HA-tagged Iporin. After lysis, cell extracts were blotted and incubated with the Aip519 and the monoclonal 16B12 anti HA antibody, respectively. The Western blot displays a signal of about 220 kDa for both antibodies. **(Ab) **Cytosol/membrane fractionation with extracts from transiently transfected HeLa cells. Recombinant HA-Iporin was detected with anti HA (16B12) antibody. Fractionation of full-length HA-Iporin and HA-Iporin ΔN847 revealed that most of the recombinant proteins were present in the membrane fraction (P100) with small amounts in the cytosolic (S100) fraction. In contrast, the transiently expressed HA-Iporin ΔC853 was present in a high concentration in the cytosolic (S100) fraction. **(Ac) **Comparison between untransfected and HA-rab1bQ67L transfected HeLa cells. Blots were analyzed using the Aip519 (Iporin), anti-GM130 (GM130), anti-p115 (p115), anti-calnexin and 1E7 (anti-rab1b) antibodies. **(B) **Immunofluorescence staining of recombinant HA-Iporin expressed in HeLa cells shows a cytosolic distribution with a perinuclear enrichment in spotted structures. Bar 10 μm.

Although the calculated mass of Iporin (deduced from the cDNA sequence) is 161,195 kDa, the endogenous protein is detected at about 220 kDa in Western blot analyses (Fig. 5Ac first row). We assume that the molecular weight shift observed in the Western blot analyses could result from posttranslational modifications within the aminoterminal part of the Iporin molecule, because transient transfections in HeLa cells with aminoterminal constructs showed the highest difference between measured and calculated weight (data not shown). However, in lysates from transfected HeLa cells expressing HA-tagged full length Iporin, the affinity-purified antibody Aip519 detected a 220 kDa protein as did the 16B12 antibody, which is directed against the HA epitop (Figure 5Aa).

To obtain information about the membrane association of Iporin, we performed cytosol/membrane fractionation and therefore we transiently transfected HeLa cells with vectors encoding full-length Iporin, an aminoterminal fragment (Iporin ΔC853) or the C-terminal fragment (Iporin ΔN847), respectively (Figure 5Ab). Transfected cells were lysed and extracts were separated by differential centrifugation steps into cytosol (S100) and membrane (P100) fractions. Equal amounts of protein were analyzed by Western blotting. Calnexin distribution was used as a marker for integral membrane proteins to verify the purity of the fractions (not shown). Our data demonstrate that full-length HA-Iporin and HA-Iporin ΔN847 seem to associate with cellular membranes or insoluble parts of cells, because major part of the proteins was detected in the P100 fractions (Figure 5Ab). Interestingly, the HA-Iporin ΔC853 mutant, which lacks the C-terminus was highly enriched in the cytosolic fraction, indicating that parts of the C-terminus play an important role in the cellular localization of Iporin (Figure 5Ab). To address the question, whether the overexpression of permanently active rab1b influences the cellular distribution of Iporin, we transiently transfected HeLa cells with vectors encoding for HA-tagged rab1b Q67L and performed further fractionations (Figure 5Ac). The comparison between non-transfected and transfected HeLa cells showed, that the overexpression of HA-rab1b Q67L had no remarkable effect on the distribution of Iporin. GM130, p115 and Calnexin were used as control proteins and showed as well no significant changes.

Hence our Aip519 polyclonal antibody was inapplicable for immunohistochemistry we transiently transfected HeLa cells with HA tagged Iporin and used the anti HA-antibody 16b12 for staining. The immunofluorescence shows a cytosolic pattern of HA-Iporin, enriched in small, brighter spots. However, most of the cells showed a faint perinuclear enriched fluorescence where usually the Golgi/ER structures are localized (Figure 5B).

### Iporin interacts with the Golgi matrix protein GM130

**Figure 6:**
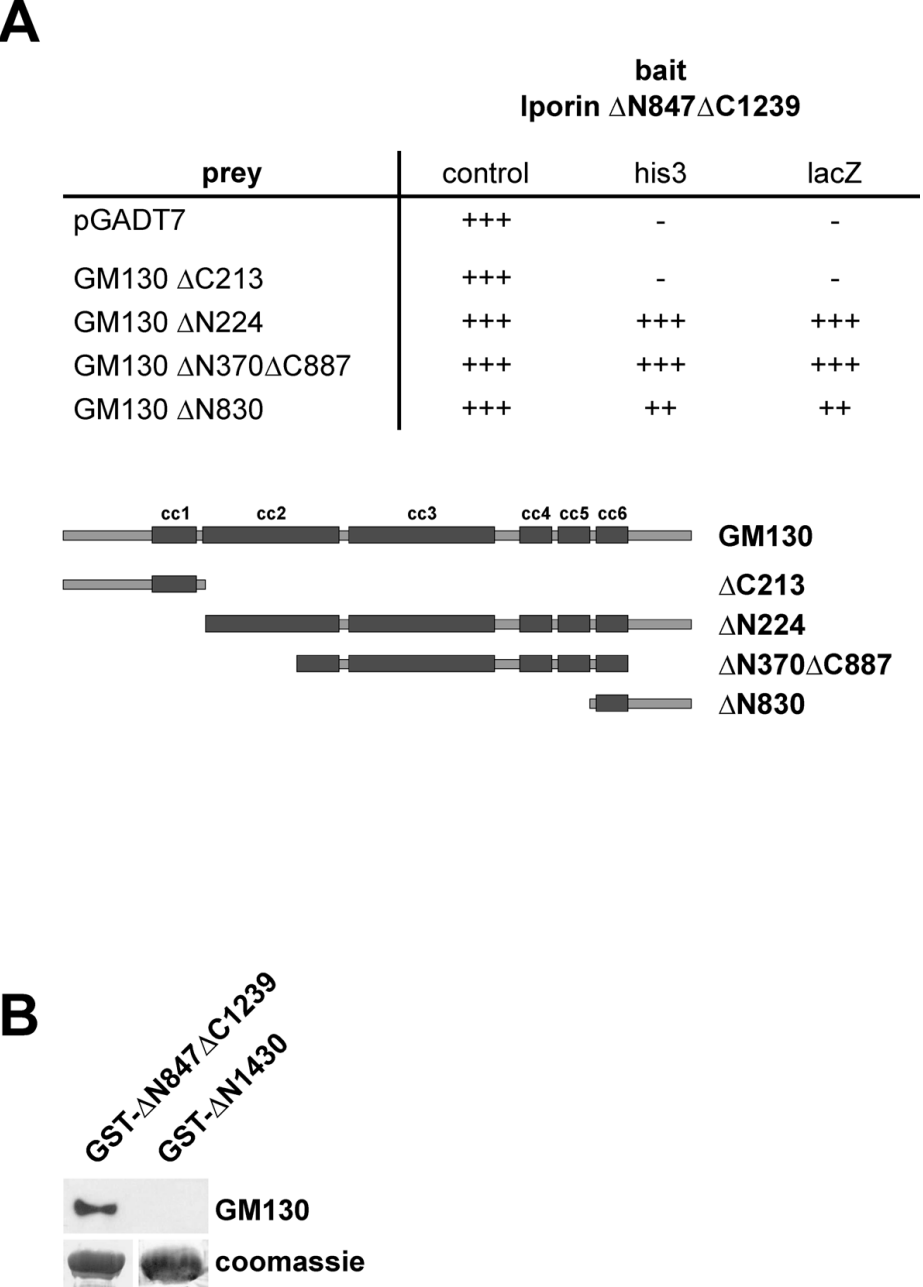
**Interaction of Iporin with GM130. (A) **Yeast co-transformations revealed an interaction between Iporin and GM130. Using different GM130 deletion mutants, we were able to map the binding site to the coiled-coil region 6 (Figure 6A). The bait construct IporinΔN847ΔC1239 was cloned into the pAS2-1 vector and the prey constructs into pGADT7. Clones were cultivated on selection plates as described previously (see Table 1). = no growth on selection plates or no β-galactosidase activity; ++ = strong growth or β-galactosidase activity appears after a few hours; +++ = very fast growth or high β-galactosidase activity **(B) **GST-pulldown of endogenous GM130 with GST-IporinΔN847ΔC1239 or GST-Iporin ΔN1430. After incubation with HeLa cell extracts, GST-IporinΔN847ΔC1239 showed an interaction with GM130, whereas GST-Iporin ΔN1430 did not bind to GM130. The lower panel shows the Coomassie blue stained blot membrane with equal amounts of GST fusion protein.

Surprisingly, yeast two hybrid co-transformation experiments showed an additional interaction between the two rab1 binding proteins Iporin and GM130. Mapping of the binding site of GM130 revealed, that the coiled-coil domain 6 is responsible for the interaction with Iporin, whereas GM130 coiled-coil domain 3 mediates the interaction with rab1b (Figure 6A) [15]. To confirm the yeast data, we performed *in vitro *binding assays. Recombinant GST-Iporin ΔN847ΔC1239 as well as GST-Iporin ΔN1430 proteins were coupled to glutathione Sepharose beads and then incubated with HeLa SS6 cell extracts, which expressed the GM130 protein in considerable amounts. After washing steps, the bound proteins were separated by SDS-page and analyzed by Western blotting analysis using GM130 antibodies. As shown in figure 6B, only GST-Iporin ΔN847ΔC1239 is able to bind to endogenous GM130, whereas the GST-IporinΔN1430 was unable to interact (Figure 6B).

## Discussion and conclusions

The cellular function of the small GTPase rab1 is not fully understood, thus the identification of novel rab1 interacting molecules may help to elucidate its role in vesicular transport. Using the yeast two-hybrid system, we were able to isolate Iporin as a new protein, which specifically interacts with the activated, GTP-bound conformation of rab1. Interestingly, Iporin also showed an interaction with the rab1 homolog from yeast Ypt1p, indicating that formation of this protein/protein complex is conserved during evolution.

Northern blot analysis of Iporin mRNA revealed an ubiquitous expression pattern (Figure 4). Highest amounts of Iporin mRNA were observed in brain and testis, suggesting that Iporin might have a distinctive role in these tissues. Neurogenesis as well as spermatogenesis are processes that result in the production of highly polarized cells and we speculate that Iporin might act as a regulator in these cellular systems [41].

Applying a set of deletion mutants and chimeras of rab1, we demonstrated that the Iporin binding domain is localized within the N-terminal half of rab1 and that the rab1 N-terminal hypervariable region is essential for the interaction (Figure 1). In contrast to PRA1, another known rab1 effector protein, our results show that the prenylation site of the rab1 GTPase is not necessary for the binding to Iporin [42]. A well characterized interaction between a rab GTPase and an effector molecule is the rab3a/rabphillin-3a interacting complex [43]. The specific interaction of rab3a with rabphillin-3a is mediated through a rab3a "pocket" contributed by three rab complementary-determining regions (RabCDR), which are localized at the N- and C-terminus. The RabCDRs are proposed to form regions of variable sequences among the rab family within a structural conserved framework and act as a structural element for protein/protein interactions [43]. This model was supported by the work of Seabra and co-workers. They defined a model of conserved regions within the rab family (RabF) and regions only conserved among the rab isoforms (RabSF) [31]. Projected on a three dimensional model of rab3a, the previously described RabCDR regions are represented by the RabSF1, 3 and 4 regions. They form one interaction surface and the RabSF2 region forms another surface, almost on the opposite side [31]. The data presented here about the interaction of the rab1b Q67R deletion mutants and the permanently active rab1b/rab6a chimeras with Iporin and Iporin Δ847, respectively, revealed that the N-terminal half of rab1b, including RabSF1 and RabSF2, is essential for the interaction with Iporin (Figure 1B). The C-terminal elements, comprising RabSF3 and RabSF4, do not play a role in the formation of the complex and may be necessary for structural reasons only. Surprisingly, this is in contrast to the results received from the rab3a/rabphillin-3a complex. Our data suggest that Iporin is able to interact with parts of both interaction surfaces. Thus, these results support the theory of a multi-protein interaction interface, where molecules can interact with different parts of both surfaces.

To date, four proteins (p115, GM130, golgin-84 and MICAL) have been identified which interact specifically with the activated conformation of rab1 [14-17,25,27,28]. GM130 is a cis-Golgi-localized coiled-coil protein, targeted to membranes via the peripheral membrane protein GRASP65 [44]. Additionally, GM130 interacts with activated rab1, rab2, rab33b, the vesicle tethering-protein p115 and as we recently observed with Iporin [15,45]. p115 has been identified to play a pivotal role in docking of COPI vesicles to the Golgi membranes by recruiting GM130 and Giantin in a long tethering complex [21,22]. Giantin is a transmembrane protein, located on COPI vesicles and shares similarities with the recently identified golgin-84 and CASP [16,46,47]. It is worth to note, that rab1 was also shown to recruit p115 during budding to the ER-compartment to program COPII vesicles for fusion [14]. Another aspect, which has been discussed to be required for tethering events at the ER-Golgi stage are large multisubunit complexes like TRAPPI (transport protein particle). TRAPPI has been identified in yeast to function as tethering factor with GEF activity (guanosine exchange factor) for Ypt1p [48,49]. The importance of this complex for mammalian transport steps between ER and Golgi remains to be clarified, but the increasing number of rab1 interacting molecules raises the theory of a huge protein complex with rab1 in a central position. Another possibility might be that activated rab1 only mediate interactions between other proteins, e.g. Iporin and GM130, in a time or place dependent manner.

Iporin displays some features that are quite different from the known rab1 interacting molecules. First GM130 as well as golgin-84 contain coiled-coil regions that are essential structural elements for the interaction with rab1 [15-17,28,50]. However, the rab1b binding domain of p115 has not been characterized so far [14,51]. Iporin contains no coiled-coil regions, but interacts via a RUN domain with rab1 (see below). Second the known rab1 effector proteins are membrane-associated proteins, which are involved in tethering processes between donor and acceptor membranes at ER-Golgi compartments [14,17,22,46]. Iporin displays a distribution (Figure 5Ab–c, 5B), which is also different from known proteins belonging to the tethering complex at Golgi membranes. Similar to MICAL, the localization of Iporin is not restricted to the ER-Golgi compartment leading to the conclusion that, in contrast to GM130 or GRASP65, Iporin is not a member of the Golgi matrix protein family [15,24,27]. The cytosol/membrane fractionation of HeLa cell extracts revealed a mainly membranous association (P100) of transiently transfected and endogenous Iporin, which is an evidence for a high and stable membranous association through its function (Figure 5Ab–c). At this step, Iporin might recruit further proteins to form a complex needed for a regulative purpose.

Interestingly, the deletion mutants Iporin ΔN847 and Iporin ΔC853 show a different distribution in the S100 fraction. Iporin ΔC853 exhibits a cytosolic accumulation, which is a hint for a much weaker accessibility to membranes (Figure 5Ab). This accumulation seems to be due to the missing carboxyterminal part of the protein and not the result of overexpression effects (Figure 5Ab). To test the influence of overexpressed permanently active rab1b, we transiently transfected HeLa cells with HA-tagged rab1bQ67L and compared transfected to non-transfected cells. The obtained data showed no significant effect on Iporin membrane association (Figure 5Ac). One could argue that the membrane association is not necessarily coupled to the interaction with rab1. Like GM130, which is linked via the GRASP65 protein to membranes, another protein, not yet identified, might be responsible for the attachment of Iporin to membranes. In addition, the overexpression of HA-rab1bQ67L had also no effect on the GM130 distribution, which interaction with rab1 is well characterized. Another reason could be, that for the interaction with rab1 only a limited amount of Iporin is sufficient and that the HA-rab1b Q67L compete with the GTP-bound endogenous rab1b.

As mentioned before, Iporin contains a RUN and a SH3 domain as well as proline- and glutamic acid-rich regions. These motifs have been described to function as targets for protein/protein interactions [35,52,53]. The RUN domain was previously shown to be part of the rap2 binding region and is supposed to mediate the interaction between the small GTPase rap2 and the effector protein RPIP8 [35]. In contrast, the RUN domain of Rabip4, an effector protein of the small GTPase rab4, has no influence on the interaction, but was supposed to be responsible for an association with a filamentous network [36]. We were able to map the rab1 interacting domain to a RUN domain-containing region and we suggest a rab1-specific interacting function for the RUN domain of Iporin (Figure 3).

Using the phage display method we identified PPΨX_2_M as a new SH3 binding motif, which is different from the originally known PXXP motif [52]. The Iporin SH3 domain together with the polyproline regions may act as regulative targets for not yet identified interacting proteins. Such a link was recently described for the small GTPase rab5. RN-tre, a rab5-specific GTPase-activating protein (GAP) interacts with Grb2. This association is mediated via the SH3 domains of Grb2 and the proline-rich regions of RN-tre [54].

Our recent discovery of an additional not yet identified interaction partner of Iporin, the GM130, is a very interesting piece of the "Golgi puzzle" (Figure 6). As has been shown in this and previous manuscripts, both proteins, the Iporin and GM130 interact with rab1 [15,17]. As mentioned above, GM130 is important for vesicle docking processes at the *cis*-Golgi, where it interacts with p115. GM130 itself is recruited to ER-derived vesicles and vesicular-tubular clusters by the rab1 GTPase [50]. One could speculate, that Iporin, while interacting with rab1b, specifies the recruitment of the incoming vesicle to the *cis*-Golgi by displaying a high affinity to GM130 by finally binding to this protein. The presented data showed that the binding site of Iporin is different from other GM130 interacting proteins. p115 binds to the N-terminus, rab1b to the coiled-coiled region 3 and GRASP65 to the C-terminus of GM130 [15,18,44]. Iporin binds to the coiled-coiled region 6 which means that they could bind in several ways e.g. at the same time, one after another, two at the same time, etc.

The multi-domain structure of Iporin and the cellular distribution suggest that this protein might act as an adapter or scaffold protein, which links GTPases to certain intracellular signal transduction pathways. Noteworthy, Iporin displays several features different from known rab1 effector proteins. Our data about the Iporin-rab1-GM130 association demonstrate once more, that the increasing number of protein/protein interactions at different stages of ER to Golgi transport implies a more complex regulated network as originally thought.

## Methods

### Yeast two-hybrid assay

For the yeast two-hybrid screen we cloned human rab1b Q67R cDNA into the pAS2-1 bait vector and transformed Y190 as recommended by the Matchmaker II™ manual (BD Biosciences Clontech, Heidelberg, Germany). The bait yeast strain was then transformed with a human placenta cDNA library (BD Biosciences Clontech, Heidelberg, Germany) as prey. In order to test the specificity of the interaction between rab1b Q67R and putative binding partners, Y190 yeast cells were co-transformed with various bait and prey constructs and incubated on selection plates at 30°C for 5–7 days as described by manufacturer's instructions (BD Biosciences Clontech, Heidelberg, Germany). A protein/protein interaction was confirmed by β-galactosidase filter lift assays using X-gal as substrate. The cDNAs of selected yeast clones were purified, transformed into *E. coli *strain DH5α and sequenced.

### Cloning of the full-length Iporin sequence and generation of deletion mutants

To clone the full-length Iporin sequence, we amplified the cDNA encoding the N-terminal region of Iporin (which is represented by the Iporin ΔC853 deletion mutant) using the KIAA 0375 cDNA (generous gift from Takahiro Nagase, Kazusa Institute, Japan) as template. We generated a primer with a 5' *Nde*I overhang containing the predicted start codon and a primer with a 3' *Eco*RI overhang beyond a single endogenous *Hind*III restriction site, which was also found in the original prey clone. The PCR product, representing the N-terminal part, was digested with *Nde*I/*Hind*III and was inserted into the eukaryotic expression vector pSV-HA [55]. In a second step, the initially isolated prey cDNA encoding the C-terminal part of Iporin, was digested with *Hind*III and was inserted into the pSV-HA-N-terminal vector. To generate deletion mutants, fragments of Iporin cDNA were amplified with specific primers containing 5' *Nde*I and 3' *Eco*RI sites and were cloned into the pAS2-1 or pGADT7 vectors. Generation of rab1b/rab6a chimeras was described previously [29]. All resulting constructs were verified by sequencing. Details are available from A. Barnekow.

### Northern blot analysis

Multiple tissue Northern Blot (OriGene, Rockville, USA) of poly (A^+^) RNA from various human tissues was hybridized with ^32^P-labeled Iporin ΔN847 cDNA using the RediPrime Nick Translation Kit (Amersham Biosciences, Freiburg, Germany) according to the manufacturer's instructions. The X-ray film was exposed for 17 h at -80°C with an amplifying screen.

### Antibodies

The polyclonal antibody Aip519 against Iporin was raised in a rabbit immunized with GST-Iporin aa 848–991 (Eurogentech, Seraing, Belgium). The antibody was affinity purified using the antigen coupled to NHS-Sepharose (Amersham Biosciences, Freiburg, Germany). The mouse monoclonal antibody 1E7 against rab1b has been described earlier [55]. Mouse monoclonal antibodies against Calnexin (anti-Calnexin), GM130 (anti-GM130), p115 (anti-p115) and the HA-tag (16B12) were purchased from BD Biosciences (Heidelberg, Germany) and BAbCO (Berkely, USA), respectively. Secondary antibodies coupled to peroxidase were purchased from Amersham Biosciences (Freiburg, Germany).

### Expression and purification of recombinant proteins

GST-Iporin ΔN847ΔC992 fusion protein, used for immunization and GST-Iporin ΔN1430 (encoding the SH3 domain) used for the phage display method (see below) were expressed in *E. coli *strain BL21 induced with 1 mM IPTG for 3 h at 30°C. The pelleted bacteria were washed, resuspended in PBS containing protease inhibitors (complete™, EDTA-free, Roche Diagnostics, Mannheim, Germany) and sonicated 10x for 10 sec. Triton X-100 was added to 1% final concentration and the lysate was incubated on ice for 30 min. After centrifugation at 16000 × g the cleared supernatant was incubated with glutathione-Sepharose beads for 30 min at 4°C. The beads were washed twice with chilled PBS and the fusion protein was eluted with 50 mM Tris-HCl, pH 8.0, containing 10 mM reduced glutathione. Samples were dialyzed against PBS overnight. The recombinant proteins used for GST-pulldown experiments were treated as described above without the elution and dialyzation step. Cleared supernatants were stored at -80°C until use.

### In vitro binding assay

GST-pulldown assays were performed using bacterially expressed GST fusion proteins containing the deletion mutants Iporin ΔN847ΔC1239, ΔN847ΔC992 and ΔN1430. Equal amounts of the GST fusion proteins or GST were immobilized on 10 μl packed glutathione-Sepharose beads and incubated overnight at 4°C. After the beads were washed three times with LB buffer (10 mM Tris pH7.4, 150 mM NaCl, 1 mM MgCl_2_, 1 mM CaCl_2_, 0.2% Triton X-100, complete™ EDTA-free, Roche Diagnostics, Mannheim, Germany), they were incubated with cytosolic extract from BHK cells, transiently transfected with pSV-HA-rab1b Q67R using the Ca-phosphate/DNA precipitation method [56]. To prepare the cytosolic extract, BHK or HeLa cells were washed three times with chilled PBS and scraped in a volume of 300 μl LB buffer. After homogenization by 15 passes through a 25 gauge needle, lysates were centrifuged at 14000 × g, 4°C for 1 h. The beads containing tubes were incubated for 3 h at 4°C on a rocking platform, supplemented with 50 μl goat serum and were adjusted with LB to a total volume of 300 μl. Beads were recovered by centrifugation, washed three times with 500 μl LB. Proteins were eluted by boiling the beads in SDS sample buffer analyzed by SDS/PAGE and Western blotting. The GM130-pulldown was performed as above with untransfected HeLa cell extracts and without the supplementation of goat serum.

### Cell lines

BHK and HeLa cells were grown in Dulbecco's modified Eagle's medium (DMEM) supplemented with 10% fetal calf serum and 1% glutamine at 37°C in a humidified 5% CO_2 _incubator.

### Cytosol/membrane fractionation

HeLa cells were grown on 10 cm tissue culture plates and transiently transfected with different pSV-HA-Iporin constructs or pSV-HA-rab1bQ67L using the Ca-phosphate/DNA precipitation method. After 20 hrs, cells were washed three times with chilled PBS, freezed at -80°C, thawed and scraped into 300 μl CMF buffer (250 mM sucrose, 10 mM Hepes/KOH pH 7.4, 1 mM EDTA, complete™ EDTA-free, Roche Diagnostics, Mannheim, Germany). Cells were homogenized by 15 passes through a 25 gauge needle and centrifuged at 1000 × g, 4°C for 2 min. The resulting PNS (postnuclear supernatant) was underlayered with 10 μl of 50% sucrose and centrifuged at 14000 × g and 4°C for 15 min. The supernatant was centrifuged for a second time at 100000 × g and 4°C for 1 h, which provided the S100 supernatant and the P100 pellet fraction. The P100 pellet was washed with 200 μl of CMF and centrifuged once more. Samples were analyzed by SDS/PAGE and Western blotting.

### Immunofluorescence analysis

HeLa cells were grown at 40–60% confluency on coverslips and transiently transfected using the Polyfect transfection reagent (Qiagen, Hilden, Germany) according to manufacturer's instructions. After 24 hrs, the cells were washed with chilled PBS and fixed for 5 min. in 100% methanol at -20°C. The fixed cells were washed in PBS and blocked for 20 min. in PBS supplemented with 10% (v/v) goat serum. Monoclonal anti-HA tag antibodies (16B12) were diluted in PBS containing 2% (v/v) goat serum and incubated with the cells for 30 min. at RT. After washing with PBS, Alexa Fluor 488-conjugated goat anti-mouse IgGs were applied for 30 min. The cells were washed again with PBS, rinsed in water and mounted in Mowiol 4–88 (Calbiochem, Bad Soden, Germany).

### Phage display

The fUSE5/15-mer M13 phage displayed random peptide library was screened with purified GST-Iporin ΔN1430 fusion protein containing the SH3 domain of Iporin. The used library consists of 2 × 10^8 ^primary clones and contains a foreign 15-mer oligopeptide on all 5 copies of pIII. The method was performed as described previously [57]. Briefly, 2 μg of purified GST-Iporin ΔN1430 fusion protein was immobilized on the surface of a 96 well plate and incubated with ~10^13 ^plaque forming units of the phage displayed random peptide library. The bound phages were washed, eluted, amplified and incubated again with newly immobilized GST-Iporin ΔN1430 fusion protein for a second and third round to purify the bound phages. The isolated phages were tested with GST as negative and GST-Iporin ΔN1430 fusion protein as positive controls by the Detection Module Recombinant Phage Antibody System (Amersham Biosciences, Freiburg, Germany) according to the manufacturer's instructions. The DNA from positive clones was isolated and sequenced.

## Abbreviations

Iporin, interacting protein of rab1; ER, endoplasmatic reticulum; Golgi, Golgi apparatus; GST, glutathione S-transferase; DMEM, Dulbecco's modified Eagle's medium; SH3, Src homology 3; RUN, RPIP8/UNC-14/NESCA; RabF1-5, rab family region 1–5; RabSF1-4, rab subfamily region 1–4; aa, amino acid; NESCA, new molecule containing SH3 at the carboxy-terminus; RPIP8, Rap2-interacting protein 8; Rabip4, Rab4 interacting protein; Rufy1, RUN and FYVE domain containing protein; SNARE, soluble n-ethylmaleimide-sensitive factor attachment protein receptor; SM, Sec1/Munc18-like proteins, MICAL, a Molecule Interacting with CasL; GM130, Golgi matrix protein with 130 kDa.

## Authors' contributions

MB: screening, yeast transformations, cloning of Iporin, construction of mutants, GST- pulldown, figure preparation, manuscript preparation

JF: construction of deletion mutant, co-transformation

JK: northern blot analysis, manuscript preparation

EO: GST-pulldown, cytosol/membrane fractionations

TM: phage display

MK: GM130 experiments

TW: conception

AB: conception, design, manuscript preparation, research funds collection
